# Intracluster correlation coefficients and coefficients of variation for perinatal outcomes from five cluster-randomised controlled trials in low and middle-income countries: results and methodological implications

**DOI:** 10.1186/1745-6215-12-151

**Published:** 2011-06-14

**Authors:** Christina Pagel, Audrey Prost, Sonia Lewycka, Sushmita Das, Tim Colbourn, Rajendra Mahapatra, Kishwar Azad, Anthony Costello, David Osrin

**Affiliations:** 1 Clinical Operational Research Unit, University College London, UK; 2 UCL Centre for International Health and Development, Institute of Child Health, University College London, UK; 3 MaiMwana Project, Mchinji, Malawi; 4 Society for Nutrition, Education and Health Action (SNEHA), Dharavi, Mumbai, India; 5 MaiKhanda, Malawi; 6 Ekjut, Chakradharpur, Jharkhand, India; 7 Perinatal Care Project, Diabetic Association of Bangladesh (BADAS), Dhaka, Bangladesh

## Abstract

**Background:**

Public health interventions are increasingly evaluated using cluster-randomised trials in which groups rather than individuals are allocated randomly to treatment and control arms. Outcomes for individuals within the same cluster are often more correlated than outcomes for individuals in different clusters. This needs to be taken into account in sample size estimations for planned trials, but most estimates of intracluster correlation for perinatal health outcomes come from hospital-based studies and may therefore not reflect outcomes in the community. In this study we report estimates for perinatal health outcomes from community-based trials to help researchers plan future evaluations.

**Methods:**

We estimated the intracluster correlation and the coefficient of variation for a range of outcomes using data from five community-based cluster randomised controlled trials in three low-income countries: India, Bangladesh and Malawi. We also performed a simulation exercise to investigate the impact of cluster size and number of clusters on the reliability of estimates of the coefficient of variation for rare outcomes.

**Results:**

Estimates of intracluster correlation for mortality outcomes were lower than those for process outcomes, with narrower confidence intervals throughout for trials with larger numbers of clusters. Estimates of intracluster correlation for maternal mortality were particularly variable with large confidence intervals. Stratified randomisation had the effect of reducing estimates of intracluster correlation. The simulation exercise showed that estimates of intracluster correlation are much less reliable for rare outcomes such as maternal mortality. The size of the cluster had a greater impact than the number of clusters on the reliability of estimates for rare outcomes.

**Conclusions:**

The breadth of intracluster correlation estimates reported here in terms of outcomes and contexts will help researchers plan future community-based public health interventions around maternal and newborn health. Our study confirms previous work finding that estimates of intracluster correlation are associated with the prevalence of the outcome of interest, the nature of the outcome of interest (mortality or behavioural) and the size and number of clusters. Estimates of intracluster correlation for maternal mortality need to be treated with caution and a range of estimates should be used in planning future trials.

## Background

Public health interventions are increasingly evaluated using cluster-randomised controlled trials (cRCTs) in which groups rather than individuals are allocated randomly to treatment and control arms. Cluster-randomised designs are pertinent to public health for three main reasons [1]. First, many contemporary public health interventions are large in scale, complex in nature, and 'unblinded' in design because they require the active involvement of participants. It is often more appropriate and feasible to implement them 'with' groups or communities rather than to apply them 'to' individuals. Second, cRCTs have the advantage of minimising the risk of contamination that would occur if individuals from the same community were randomised to different treatment arms. Third, cRCTs enable policy-makers and researchers to assess the population-level effects of an intervention applied to a proportion of a population, giving more information about its effectiveness in real life settings. There are now numerous examples of well-conducted public health cRCTs from high, middle and low-income countries. The Mwanza study, for example, tested the impact of improved sexually transmitted infection (STI) case management on the incidence of HIV infection in Mwanza, Tanzania, by randomising 12 pair-matched communities to intervention or control arms [2]. Other, current cRCTs include a UK study in which deprived areas of London have been randomised to receive interventions promoting healthy eating, physical activity and mental health [3], and, in India, a trial in which urban wards of Mumbai were randomly allocated to a community mobilisation intervention for improved maternal and newborn health [4].

An important implication of cluster-randomised designs is that intervention recipients are often groups of individuals who share socio-economic and cultural characteristics and have similar health outcomes by virtue of living in the same area. Similarity between individuals in the same cluster lessens the variability of responses within clusters (within-cluster variance), thereby artificially magnifying differences in outcomes between clusters and reducing the power of trials to detect true differences between intervention and control arms. An intracluster correlation coefficient (ICC) is commonly used to quantify how much more similar outcomes are for individuals within clusters than for those in different clusters [5,6]. The ICC is defined as the ratio of the between-cluster variance to the total variance (both between and within clusters), and therefore has a value between 0 and 1. An ICC of 0 indicates that individuals within clusters are no more similar to each other than individuals from different clusters (there is no between-cluster variability), while an ICC of 1 indicates that individuals within the same cluster all have identical outcomes (there is no within-cluster variability) [1].

The increase in variance due to clustering, or design effect, is given by **1 **+ (*m*-**1**) *ICC*, where *m *is the average cluster size [7]. Within-cluster correlation has a correlate - between-cluster variation - which is commonly expressed as the coefficient of variation, *k*. For binary outcomes, the relationship between the ICC and *k *has been defined as , where π is the probability of the binary outcome of interest [8]. Obtaining estimates of ICC or *k *is essential to calculate sample sizes for studies with clustered designs, but these are seldom available to researchers when designing trials and are often approximated from previous research or through modeling techniques [9-11].

Several community-based interventions to reduce mortality in mothers and neonates in low-resource settings have recently been evaluated through cRCTs, and there is increasing momentum to support health systems in delivering components of these community programmes at scale [12-17]. Designing adequately powered studies to measure the impact of programmes will be critical. However, most ICC or *k *estimates for perinatal health outcomes available in the literature come from hospital-based studies and may therefore not reflect outcomes in the community [18]. In this study, we report ICC and *k *estimates for perinatal health outcomes from community-based trials in three countries (Bangladesh, India, and Malawi) to help researchers planning efficacy and effectiveness evaluations of interventions to improve maternal and newborn health.

## Methods

**Figure 1:**
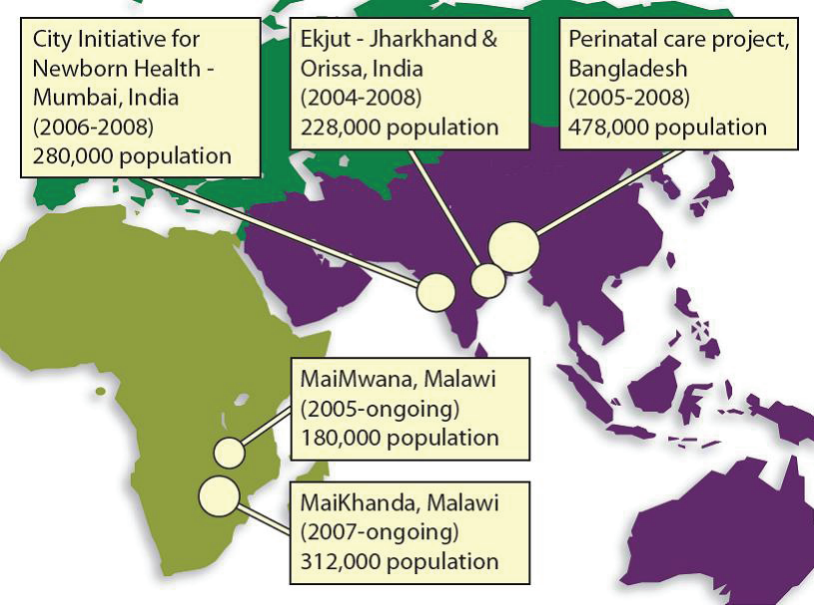
**Location, population size and duration of studies**.

We estimated ICC and *k *for a range of outcomes using data from five community-based cRCTs, three of which have not yet reported final results (MaiMwana and MaiKhanda in Malawi, and the City Initiative for Newborn Health in India). All five trials sought to evaluate community mobilisation interventions with women's groups to improve maternal and newborn health outcomes. A version of this intervention was first tested in Nepal, where it achieved a 30% reduction in neonatal mortality [18]. Figure 1 shows the location of the trials included in the study. Table 1 describes their design, sample size, and the number and characteristics of study clusters.

**Table 1:** Characteristics of studies used, prevalence, and rates, for key perinatal indicators from 5 community-based cluster RCTs

Project Country	Perinatal Care Project Rural Bangladesh	Ekjut Rural India	City Initiative for Newborn Health Urban India	MaiMwana Malawi	MaiKhanda* Malawi
Study location	Three districts: Bogra, Maulvibazaar and Faridpur	Three districts of Jharkhand and Orissa: Keonjhar, West Singhbhum and Saraikela	Mumbai municipality	Mchinji district	Three districts: Lilongwe, Salima and Kasungu
Period for which data are included	1^st ^Feb 2005 - 31^st ^Dec 2007	1^st ^July 2005 - 30^th ^June 2008	1^st ^October 2005 - 30^th ^September 2008	1^st ^January 2005 - 31^st ^January 2009 (study is ongoing)	1^st ^July 2008 - 31st July 2010 (study is ongoing)
Estimated population	478 000	228 000	280 000	180 000	312 000
Design	Two-by-two factorial cluster RCT	Cluster RCT	Cluster RCT	Two-by-two factorial cluster RCT	Two-by-two factorial cluster RCT
Stratification	By district (3 strata)	By district (3 strata)	By municipal ward (6 strata)	None	None
Cluster characteristics	Villages making up a union	8-10 village with residents classified as tribal or OBC	1000-1500 households in slum areas	Aggregated villages and group village headman areas	Aggregated villages and group village headman areas in the catchment area of one Health Centre/Dispensary
Total number of clusters (Number included in this study)**	18 (5)	36 (18)	48 (24)	48 (12)	76 (30)
Annual births per cluster: Mean (SD)	587 (123)	171 (38)	131 (61)	139 (25)	143 (61)
Mean cluster population (SD, min, max)	27953 (5953, 15441-35110)	6338 (2101, 3605-7467)	5865 (1077, 4310-7750)	3958 (404, 3068-4645)	3934 (1332, 2121-8558)
Crude birth rate***	20.8	28.1	22.3	35.1	35.0

* MaiKhanda data are provisional as verification of deaths and follow-up of missing women are still ongoing.

**These are 'pure' control clusters. In the case of factorial designs, none of the interventions tested was being implemented in these clusters.

*** Number of live births per 1000 population during study period. We chose to use population estimates at the mid-point of trials as the denominator.

We report ICC and *k *for seven selected perinatal health outcomes: neonatal mortality, stillbirths, maternal mortality, four or more antenatal care visits, skilled birth attendance (by a nurse or doctor), exclusive breastfeeding prior to interview at about six weeks, and postnatal check-ups by a nurse or doctor in the same period. In addition, we calculated ICC and *k *for uptake of HIV testing and use of insecticide treated bed nets in one site in Malawi, as these are important outcomes for African studies. We selected stillbirths and neonatal mortality because they are outcomes commonly used to calculate perinatal study sample sizes, maternal mortality because there are few reports of ICC or *k *since it is a rare outcome, and other outcomes as process indicators that may be increasingly used in the future to calculate sample sizes for effectiveness studies. We used the framework proposed by Campbell and colleagues [19] to report ICC and provide: a description of the dataset from which estimates were drawn and a description of the outcome characteristics; information on how ICC was calculated; and information on its precision through a 95% confidence interval.

We chose to exclude data from the intervention clusters of the five trials from the main calculations of ICC and *k *for two reasons. First, three of the five trials had not yet reported results for their primary outcome (neonatal mortality) at the time of analysis. Second, we hypothesised that the interventions tested in these studies could influence ICC for perinatal outcomes, as they sought to increase care-seeking and institutional deliveries. We felt that discussing the extent and reasons for variation between trial intervention and control clusters was beyond the scope of this paper and therefore opted to exclude data from the trial intervention clusters. However, recent research underscores the importance of taking into account the likely effect of the intervention when estimating the between-cluster variability [20].

### Data collection

In all five trials perinatal outcomes were measured using community-based prospective surveillance systems [4]. These systems differed slightly between sites and are briefly described here.

The Perinatal Care Project trial took place in three rural districts of Bangladesh. In each district, six unions per district (mean union population: 27 953) were purposively sampled for surveillance. In these unions, Traditional Birth Attendants (TBAs) prospectively reported births and deaths to women in pregnancy or up to six weeks after delivery within an area covering around 200 households. Interviewers then verified the births and deaths, paid the TBA an incentive for each correct identification, and completed a questionnaire covering maternal background characteristics, the antenatal, delivery, and post-partum periods with the mother, or, in case of a maternal death, with a relative. All eligible women identified were also asked if they could identify any other pregnant women.

In the Ekjut trial (Jharkhand & Orissa, rural eastern India), a key informant, usually a community member, covered around 250 households and prospectively reported any births, maternal or newborn deaths and deaths to women aged 15-49 within her allocated area. The trial area covered 36 clusters over three districts (mean cluster population 6338). Clusters were purposively sampled to include a high proportion of tribal communities. In each district, 12 interviewers met with key informants, verified all births, and paid the key informant a fee for every correct identification. The interviewers administered a structured questionnaire to mothers at around six weeks after delivery to collect information about their socio-demographic characteristics and events during the antenatal, delivery and postnatal periods.

The City Initiative for Newborn Health in Mumbai monitored 48 urban slum clusters of about 1000 households each (5500 population), selected randomly from a sampling frame of 92 clusters over six municipal wards. Births and deaths were identified by 99 locally resident women, generally two per cluster, each covering an average 500 households and receiving a fee for identification. Events were confirmed by 12 interviewers, each responsible for four clusters, who visited mothers at home and arranged follow-up interviews.

In the MaiMwana trial (Malawi), pregnancies were prospectively identified by a paid woman enumerator who visited all females aged 10-49 years once a month to ask about missed menses. Information was recorded in a register of all women of childbearing age and updated monthly. Live births were followed up with interviews one month and six months after birth [23]. There were 48 women enumerators (one per cluster), and 48 interviewers (also one per cluster) who verified births and conducted post-partum interviews.

In the MaiKhanda evaluation, also in Malawi, the team selected health centre catchment areas with a typical population of 30 000. Within each of these, a population of 4000 was selected for surveillance by randomly choosing catchment populations of government community health workers that summed to roughly 4000. In each of these areas, 20-25 key informants reported data on births, deaths, and the antenatal and postnatal period. In all sites, verbal autopsies were conducted for neonatal deaths, stillbirths, and maternal deaths.

### Trial designs

The trials used moderately different designs to arrive at one-to-one random allocation of intervention and control clusters. In the Perinatal Care Project (Bangladesh) and the Ekjut trial (rural India), districts were identified and clusters purposively sampled, then randomised within each district. In the Bangladesh Perinatal Care Project, clusters initially randomised to the women's group interventions were also re-randomised to receive training for Traditional Birth Attendants in a two-by-two factorial design. In the Mumbai City Initiative for Newborn Health trial, urban slum clusters were randomly sampled from a frame stratified by six purposively selected municipal wards, and then randomly allocated to the intervention. MaiKhanda and MaiMwana in Malawi used two-by-two factorial designs in which clusters were allocated randomly to a women's group intervention or no intervention, and each group was stratified by the presence or absence of another intervention - a breastfeeding counseling intervention in the case of MaiMwana, or a Quality Improvement of maternal and neonatal facility-based healthcare intervention in the case of MaiKhanda. For the Bangladesh Perinatal Care Project and the Malawi MaiKhanda and MaiMwana trials, we therefore only included in this study the clusters that were 'pure' controls. Details of stratification and number of clusters included are given in Table 1. Although all five trials included in the study had baseline data, we only included these for the 'pure' control clusters of the MaiKhanda study in Malawi because it started later than the other trials and had fewer cases to contribute to the analysis. One of the other trials (PCP) had retrospective baseline data, which we decided not to include in order to preserve data quality.

### Ethical approval

All trials from which data for this study were drawn were approved by the ethics committee of the Institute of Child Health and Great Ormond Street Hospital for Children (UK) and by the following research ethics committees: the ethics committee of the Diabetic Association of Bangladesh (Perinatal Care Project, Bangladesh Diabetes Somity or BADAS); an independent ethics committee in Jamshedpur, India (Ekjut trial); the Mumbai Independent Ethics Committee for Research on Human Subjects (City Initiative for Newborn Health trial, reference IEC/06/31); the Malawi National Health Sciences Research Committee (MaiMwana trial, reference MED/4/36/1/167, MaiKhanda protocol #420). All trials were conducted in disadvantaged areas with high levels of female illiteracy; all participants gave consent in writing, by thumbprint or verbally.

### Outcome definitions

We used the ICD 10 definitions of neonatal death, stillbirth and maternal death [24]:

**Stillbirth **- "A stillbirth or foetal death is a death prior to the complete expulsion or extraction from its mother of a product of conception, irrespective of the duration of pregnancy; the death is indicated by the fact that after such separation the foetus does not breathe or show any other evidence of life, such as beating of the heart, pulsation of the umbilical cord or definite movement of voluntary muscles." ICD classifies late foetal deaths (birthweight greater than 1000 gm or after 28 weeks) and early foetal deaths (500 to 1000 gm or 22-28 weeks). We use the ICD-10 criteria for stillbirth modified to include births after 28 completed weeks rather than 22 weeks.

**Neonatal death**: death within the first 28 days of an infant after "the complete expulsion or extraction from its mother of a product of conception, irrespective of the duration of the pregnancy, which after such separation, breathes or shows any other evidence of life, such as beating of the heart, pulsation of the umbilical cord, or definite movement of voluntary muscles, whether or not the umbilical cord has been cut or the placenta is attached."

**Maternal death**: "the death of a woman while pregnant or within 42 days of termination of pregnancy, irrespective of the duration and site of the pregnancy, from any cause related to or aggravated by the pregnancy or its management but not from accidental or incidental causes."

For postnatal outcomes (exclusive breastfeeding and postnatal check-up by a doctor or a nurse), we used data collected between one month and six weeks after birth, except in MaiMwana Malawi where data were available on exclusive breastfeeding up to six months.

### Statistical analysis and calculations

We used two methods for estimating ICC: one-way analyses of variance in STATA (Versions 10 and 11; Stata Corp., College Station, TX) using the loneway command as described by Hayes and Moulton [1], and estimation equations from Donner and Klar [25], p84-5. We calculated estimates and confidence intervals for ICC within each stratum, then calculated the unweighted average of the ICC estimates and the lower and upper bounds of the confidence intervals across strata. For the ICC estimates calculated using Donner and Klar's equations, we calculated 95% confidence intervals using Fisher's method [26], which uses the following equation:

 where

 where *m *is the mean number of births per cluster and *n *is the number of clusters in the control arm. Where the estimate for the lower bound of the confidence interval for ICC was negative, we set it to zero, assuming that the population within our clusters would not be more heterogeneous than the entire study population.

For all outcomes, we calculated *k *from the intracluster correlation coefficients by reversing the equation from Hayes and Moulton [1]: , where ICC is the estimated intracluster correlation coefficient and  is the observed proportion of the binary outcome for the entire population.

Several practical issues arose when calculating ICC using individual-level datasets in which the denominators were births. For example, we recoded the vital status for mothers who died but had twins so that maternal deaths were not double-counted. In addition, calculating ICC for maternal mortality (where live births are the denominator) required adding antepartum deaths to the list of live births so that they could be counted.

### Simulation experiments

In order to explore further the impact of cluster size and number on the reliability of estimates of *k*, we ran two simple simulations for maternal and neonatal mortality in Microsoft Excel 2003 (Microsoft Corporation). In each simulation we considered a hypothetical study area with a given number of clusters and a given number of live births per cluster. We set the maternal mortality ratio (MMR) at 300 deaths per 100 000 live births and the neonatal mortality rate (NMR) at 40 deaths per 1000 live births. For each run of the simulation, we randomly assigned neonatal and maternal outcomes for each cluster by generating random numbers and assigning outcomes according to the assumed mortality rates. By doing this, we made an explicit assumption that the outcomes of births were independent of the outcomes of other births, so that the coefficient of variation, k, for each simulation was identical to zero (individuals within clusters were set to be no more similar to each other than to individuals from different clusters). Each run of the simulation thus generated a hypothetical set of maternal and neonatal outcomes, from which we calculated the coefficient of variation *k *that would be estimated if these were real data, using the formula described by Hayes and Moulton [1, p.18]. The impact of cluster size and number of clusters is evaluated by comparing estimates of *k *to the 'true' *k *of zero: the smaller the estimates and the narrower their range across simulations, the more reliable the estimates.

## Results

### Prevalence and rates for selected perinatal indicators

Table 2 shows the mortality rates or outcome prevalence for our selected perinatal indicators within the five community-based samples, as well as corresponding estimates of ICC and *k*. We observed substantial differences in maternal and neonatal mortality rates between the study sites: the rural Indian setting (Ekjut trial, Jharkhand and Orissa) had the highest maternal and neonatal mortality rates, while the urban Indian setting (City Initiative for Newborn Health, Mumbai) had the lowest. In some cases, we found large differences between our community-based mortality estimates and national Demographic and Health Survey (DHS) figures. In eastern India, among rural tribal communities, the NMR was 59.1, compared with 39 per 1000 reported as the state estimate for Jharkhand in the 2007 National Family Health Survey [27]. In both Malawian studies we found lower maternal mortality rates than the most recently reported DHS figure of 984 per 100 000 [28]. These differences may reflect national and regional differences, different sample compositions, different time frames or methodological factors. We also found differences in key antenatal and care-seeking indicators between the study sites. Access to four antenatal care visits was low in all sites except Mumbai, reflecting greater access to private and public health services in urban areas. The prevalence of skilled birth attendance among mothers in Mumbai slums was four times that among mothers in eastern Indian tribal communities, and was much higher in rural Malawian than rural Asian settings.

**Table 2:** Intracluster correlation coefficients and coefficients of variation for key perinatal indicators

Project Country	Perinatal Care Project Rural Bangladesh	Ekjut Rural India	City Initiative for Newborn Health Urban India	MaiMwana Malawi	MaiKhanda* Malawi
**Neonatal mortality**					
Neonatal deaths	314	518	127	187	357
Live births	8503	8819	8283	6688	12499
Neonatal deaths, % of live births	3.7	5.9	1.5	2.8	2.9
Neonatal mortality rate, per 1000 live births	37.0	58.7	15.3	28.0	28.6
Stata-one way ICC stratum-averaged (95% CI)	0.00055 (0-0.00316)	0.00099 (0-0.00591)	-	0.00247 (0-0.00605)	0.00034 (0-0.00346)
Stata-one way *k*	0.15	0.13	-	0.29	0.15
Donner and Klar ICC (95% CI)	0.00055(0-0.0024)	0.00099 (0-0.00442)	0.0004 (0-0.0041)	0.00309 (0-0.0070)	0.00094 (0-0.00263)
Donner and Klar *k*	0.12	0.13	0.16	0.33	0.18
**Stillbirths**					
Stillbirths	298	270	106	116	406
Births	8801	9089	9719	6804	12905
Stillbirths, % of births	3.4	3.0	1.1	1.7	3.1
Stillbirth rate, per 1000 births	33.9	29.7	10.9	17.0	31.5
Stata-one way stratum-averaged ICC (95% CI)	0.00000 (0-0.00224)	0.00012 (0-0.00307)	-	0.00000 (0-0.00148)	0.00206 (0-0.00590)
Stata one way *k*	0.00	0.06	-	0.00	0.25
Donner and Klar ICC (95% CI)	0.00000 (0-0.00062)	0.00012 (0-0.00250)	0.0013 (0-0.0055)	0.000 (0-0.0015)	0.00242 (0.00002-0.00482)
Donner and Klar *k*	0	0.06	0.34	0.00	0.27
**Maternal mortality**					
Maternal deaths	19	68	21	32	42
Livebirths	8503	8819	8283	6710	12499
Maternal deaths, % of live births	0.22	0.77	0.25	0.48	0.33
Maternal mortality ratio, per 100 000 livebirths	223.4	771.1	219.8	476.9	336
Stata-one way ICC (95% CI)	0.00008 (0-0.00014)	0.00051 (0-0.00382)	-	0.00000 (0-0.00150)	0.00031 (0, 0.00172)
Stata-one way *k*	0.19	0.26	-	0.00	0.10
Donner and Klar ICC (95% CI)	0.00005 (0-0.00082)	0.00071 (0-0.00383).	0.0034 (0-0.010)	0.000 (0-0.0015)	0.00333 (0.00044-0.00622)
Donner and Klar *k*	0.16	0.30	1.16	0.00	0.99
**Four antenatal check-ups (ANC)**					
Mother received 4 or more ANC check-ups %, (N)	15.1% (8189)	23.4% (8867)	84.2% (7834)	26.6% (6436)	Not collected
Stratum-averaged ICC (95% CI)	0.04849 (0-0.18522)	0.15444 (0.30298-0.31837)	0.0211 (0-0.06073)	0.03010 (0.00398-0.05623)	-
*K*	0.52	0.71	0.06	0.29	-
**Skilled birth attendance**					
Births attended by a nurse or doctor % (N)	15.2% (8801)	23.3% (9089)	87% (7834)	41.9% (6788)	52.9% (12853)
Stratum-averaged ICC (95% CI)	0.03233 (0.00831-0.00319)	0.04103 (0.00594-0.14733)	0.02522 (0-0.07051)	0.15243 (0.04186-0.26300)	0.12699 (0.06411, 0.18926)
*K*	0.42	0.36	0.06	0.46	0.34
**Postnatal check-up**					
Infants received a postnatal check-up % (N)	20.9% (8128)	6.3% (8301)	56.5% (7711)	30.9% (5949)	-
Stratum-averaged ICC (95% CI)	0.03558 (0-0.12604)	0.01873 (0-0.05687)	0.01566 (0-0.04627)	0.23066 (0.07948-0.38185)	-
*K*	0.36	0.52	0.11	0.72	-
**Exclusive breastfeeding (1**^**st **^**6 weeks)**					
Infants exclusively breastfed for the first 6 weeks % (N)	62.6% (8128)	62.3% (8301)	64.7% (7711)	9.2% (3749)**	-
Stratum-averaged ICC (95% CI)	0.01286 (0-0.04821)	0.09163 (0.0006-0.23459)	0.01341 (0-0.04062)	0.03746 (0.00351-0.07140)	-
*K*	0.08	0.23	0.09	0.61	-
**Uptake of antenatal HIV testing**					
Uptake of HIV testing % (N)	-	-	-	39.2% (6624)	-
ICC (95% CI)	-	-	-	0.05457 (0.00950-0.09964)	-
*K*	-	-	-	0.29	-
**Use of insecticide-treated bednets during pregnancy**					
Use of insecticide-treated bednets & (N)	-	-	-	48.1% (6678)	-
ICC (95% CI)		-	-	0.06059 (0.01105-0.11013)	-
*K*		-	-	0.26	-

ICC: intracluster correlation coefficient; *k*: coefficient of variation.

* MaiKhanda data are provisional as verification of deaths and follow-up of missing women are still ongoing.

** MaiMwana collected data on exclusive breastfeeding for the first 6 months of life.

### Intracluster correlation coefficients for selected perinatal indicators

Overall, ICCs for mortality outcomes were lower than for process outcomes such as skilled birth attendance (SBA). Estimates of intracluster correlation for MMR and NMR were low in all sites. Estimates for maternal mortality were particularly unreliable, reflected in the broader confidence intervals, with a single maternal death having large impacts on ICC and *k *values. We noted that the ICCs estimated using the loneway command (one-way analysis of variance) in STATA were very similar to those calculated using the equations of Donner and Klar, especially for the more common outcomes of neonatal death and stillbirth.

ICCs for behavioural outcomes such as care-seeking were higher than those for mortality outcomes, not only because the proportions of interest were higher, but also perhaps because one might expect them to be more similar for women living in the same area than for women in other areas. It is also possible that cluster-specific features (for example, the presence of a popular health facility) could influence the proportion of outcomes such as skilled birth attendance in each cluster, and thereby ICC and *k*.

It is likely that there were genuine differences in population heterogeneity across sites. For instance, urban slums are home to a range of communities that maintain to some degree patterns of behaviour imported from their places of origin; they also maintain their rural-urban links. Urban areas might therefore be expected to be more heterogeneous than rural villages. Equally, there might be cultural factors that lead women to care for their infants more or less similarly in different countries.

Areas with smaller cluster sizes tended to yield higher ICCs. In the MaiKhanda study in Malawi, the average cluster size in Lilongwe was about three-quarters that of the other two districts, and the ICC for Lilongwe was higher. Not surprisingly, estimates drawn from larger numbers of clusters were more reliable (narrower confidence intervals) than those from small numbers. Estimates drawn from the Bangladesh Perinatal Care Project varied widely between districts and overall estimates appeared to have more face validity than district-specific data, which yielded more extreme values. This was especially the case for the maternal mortality estimates. Stratification reduced ICC as well as *k *values, but had a greater impact on ICCs. For instance, in the rural India trial, the estimate for ICC based on the average of estimates for ICC in each stratum for stillbirths was 0.00012, compared with 0.00033 when stratification was ignored.

### Measuring ICCs for rare outcomes: simulation results

Neonatal and maternal deaths are relatively rare population events. A typical MMR in a low-income setting might be around 300 per 100 000 live births. If the total number of live births per cluster over the period of a trial is 400 to 2000, there will, on average, only be between 1 and 6 deaths per cluster. Given such small numbers, we would expect much variation in the actual number of deaths reported in each cluster and, consequently, significant random variation in estimates of intracluster correlation. This is shown with our estimate of ICCs for observed maternal mortality in Table 2.

**Figure 2:**
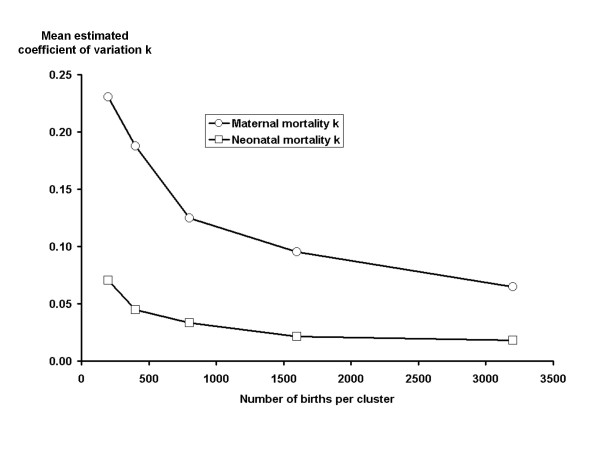
**Simulated impact of number of live births per cluster on estimated coefficient of variation (*k*) for maternal and neonatal mortality**. These simulations were run assuming that all birth outcomes were independent, i.e. the true coefficient of variation was set to zero. As the number of births per cluster increases, the estimated coefficient of variation falls closer to zero indicating that more births per cluster lead to greater reliability in estimates of the coefficient of variation. Throughout, the estimated coefficient of variation is higher for maternal mortality than for neonatal mortality.

**Figure 3:**
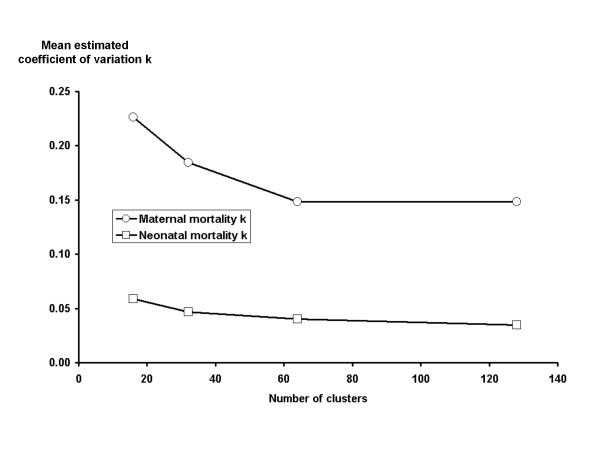
**Simulated impact of number of clusters on estimated coefficient of variation (*k*) for maternal and neonatal mortality**. These simulations were run assuming that all birth outcomes were independent, i.e. the true coefficient of variation was set to zero. As the number of clusters increases, the estimated coefficient of variation falls closer to zero indicating that more clusters lead to greater reliability in estimates of the coefficient of variation. Throughout, the estimated coefficient of variation is higher for maternal mortality than for neonatal mortality. In comparison with figure 2, the number of births per cluster has a greater impact on estimates of coefficient of variation than the number of clusters.

We ran two simulations in order to explore the impact of different numbers of live births per cluster (simulation 1) and of the number of clusters (simulation 2) on estimated *k *for maternal and neonatal mortality. Results are shown in table 3 and summarised for *k *in figures 2 and 3. In simulation 1 the MMR was fixed at 300, the NMR at 40 and the number of clusters at 32. We performed sets of 500 simulation runs, each set of runs corresponding to different numbers of live births per cluster, and calculated the mean and 75th percentile of the estimated *k *for maternal and neonatal death. In simulation 2 the number of clusters was varied. The MMR was fixed at 300, the NMR at 40 and the number of births per cluster at 400. Since the true value of *k *was zero, the results help us to understand the impact of cluster size and number of clusters on the reliability and stability of our measured point estimates for both maternal and neonatal mortality.

**Table 3:** Simulation results showing the impact of number of live births per cluster and number of clusters on k estimates for rare outcomes

Live births per cluster	Simulation mean *k *for maternal mortality	75^th ^percentile of estimated *k *for maternal mortality	Simulation mean *k *for neonatal mortality	75th percentile of estimated *k *for neonatal mortality
200	0.23	0.44	0.070	0.14
400	0.19	0.37	0.045	0.090
800	0.12	0.26	0.034	0.071
1600	0.10	0.20	0.021	0.042
3200	0.06	0.13	0.018	0.036
**Clusters**				
16	0.23	0.42	0.059	0.12
32	0.18	0.37	0.047	0.092
64	0.15	0.28	0.040	0.084
128	0.15	0.28	0.035	0.072

Figures 2 and 3 show that estimates of *k *are much less reliable when the number of births per cluster is less than 500 for both maternal and neonatal outcomes. The number of clusters was less important in determining the reliability of estimates of *k *for neonatal mortality, but for maternal mortality fewer than 60 clusters led to less reliable estimates of *k*. Across all simulations, estimates of *k *for maternal mortality were much less reliable than for neonatal mortality. Considering the 75^th ^percentile of estimates in table 3, 25% of simulation runs for maternal mortality resulted in high estimates of *k *(> 0.25), even with 800 births per cluster in simulation 1 and across all numbers of clusters in simulation 2. Estimates of *k *for neonatal mortality had a smaller range throughout.

## Discussion

Data from our trials accord with the rare published ICCs for perinatal outcomes from other community-based studies. A recent systematic review of community-based interventions to reduce maternal mortality found five cluster-randomised controlled trials seeking to improve perinatal survival [29]. None of these adjusted for cluster effect on maternal mortality and the ICC for maternal mortality was estimated to be close to 0.

For neonatal mortality, the Newhints trial in Ghana reported a baseline ICC of 0.0007256 (with a baseline neonatal mortality rate of 31 per 1000 livebirths) [30], and the Makwanpur Nepal cRCT an ICC of 0.00644 (95% CI 0.00004-0.0128) [5]. By contrast, estimates from our community-based samples were generally lower than those reported in an analysis of ICCs for perinatal outcomes in hospitals conducted as part of the 2005 WHO Survey on Maternal and Perinatal Health using data from over 90 000 births in 120 facilities across eight Latin American countries. Although the comparability of data from the WHO survey and ours is compromised by differences in data collection and outcome ascertainment methods, it is worth noting that the median ICC for neonatal death at hospital discharge or 7 days after delivery was 0.005, and the median ICC for maternal death was 0.003 in the WHO study, while our one-way analysis of variance ICC estimates range from 0.00034 to 0.00247 for neonatal death and from 0 to 0.00051 for maternal death. The higher ICCs found in the WHO study may reflect greater similarities in outcomes and practices among women who deliver in institutions, as well as differences inherent to the Latin American context.

Previous studies have suggested that the research setting (for example, primary or secondary care) and the type of variable (process or outcome) are key determinants of ICCs, but that the effects of outcome prevalence and cluster size are less straightforward [31]. Our study confirms the first two findings, but also suggests, as simulation work shows, that cluster size and the number of clusters also affect estimates of ICCs (or *k*). The simulation results highlight the difficulty in interpreting estimates of intracluster variability for rare outcomes such as maternal mortality. In almost all the simulation runs 25% of estimates of *k *for maternal mortality were greater than 0.25. Given that in these simulations there was no intracluster correlation, such estimates would have been misleading if derived from an actual data set. Given the wide range of maternal mortality estimates of *k *over the sets of 500 simulation runs, we must be cautious in placing too much trust in estimates of *k *(or ICC) for maternal mortality from individual trials, including our estimates given in table 2. However, the simulation experiments show much lower estimates of *k *for neonatal mortality with a much narrower range, indicating that estimates for NMR from individual trials are likely to be reliable, even with small numbers of clusters or births per cluster.

### Limitations

Our study had three main limitations. First, we drew upon heterogeneous trials with different cluster recruitment strategies and degrees of stratification. These design features need to be taken into consideration when using our ICCs to calculate sample sizes. For example, increased stratification leads to lower ICCs and *k *values. Second, ICCs could have been influenced by different reporting preferences in different clusters. For example, some cluster-based key informants or interviewers might have been more adept at reporting or tracking births and deaths than others. Quality of data collection (in terms of both completeness and accuracy) will strongly influence ICCs and is likely to vary between clusters given differences in fieldworkers, terrain and supervision. Finally, we used data from trials with fewer than 40 clusters in their control arms. Donner and Klar advise against overestimating the stability of a sample estimate obtained from a trial involving less than about 40 clusters. The small number of clusters selected would also have increased the confidence intervals around our ICC estimates. We also note that Fisher's method for estimating confidence intervals is unlikely to be strictly valid for the smaller samples used in this study. However, combined, our study offers the largest number of ICCs available to date for perinatal outcomes for community-based samples.

## Conclusions

Our study has three main implications. The first relates to the importance of using data drawn from community-based samples when planning effectiveness studies. Mortality estimates from our community-based trials were often substantially different from national DHS estimates and ICCs from our community-based samples in Asia and Africa were lower than those estimated from hospital-based samples in Latin America. This underscores the importance of collecting baseline data to obtain adequate estimates of mortality when planning evaluations of community interventions. Second, our results accord with findings from previous methodological studies regarding the key determinants of the intracluster correlation coefficient: the size of the ICC is related to the prevalence of the outcome of interest, and point estimates of ICC for rare outcomes such as maternal mortality are not likely to be reliable. When planning future trials, published estimates of ICCs from larger clusters are probably safer to use and a range of possible ICCs should be used for sample size calculations. Finally, as maternal and neonatal survival increases and research moves to testing interventions seeking to improve care-seeking or process outcomes, the high ICCs found for these will need to be accounted for in study sample sizes.

## List of Abbreviations used

cRCT: Cluster randomised controlled trial; DHS: Demographic and health survey; ICC: Intracluster correlation coefficient ICD: International classification of diseases; MMR: Maternal mortality ratio; NFHS: National family health survey; NMR: Neonatal mortality rate; PCP: Perinatal Care Project; SBA: Skilled birth attendant; SNEHA: Society for Nutrition, Education and Health Action; TBA: Traditional birth attendant.

## Competing interests

The authors declare that they have no competing interests.

## Authors' contributions

DO, CP and AP had the original idea for the study. CP wrote the simulation. All authors participated in data gathering, data analysis and interpretation and commented on drafts of the manuscript. AP and CP wrote the first draft and collated subsequent inputs. All authors read and approved the final manuscript.
